# Genome Sequence Announcement of Lactobacillus vaginalis LMG S-26419, Isolated from a Healthy Woman

**DOI:** 10.1128/MRA.01534-18

**Published:** 2019-02-21

**Authors:** Edoardo Puglisi, Serena Galletti, Valeria Sagheddu, Marina Elli

**Affiliations:** a Department for Sustainable Food Processes, Università Cattolica del Sacro Cuore, Piacenza, Italy; b AAT-Advanced Analytical Technologies, Fiorenzuola d’Arda, Piacenza, Italy; Broad Institute of MIT and Harvard University

## Abstract

The Lactobacillus vaginalis LMG S-26419 strain, also named CBA-L88 (BV2), was isolated at the AAT-Advanced Analytical Technologies laboratories from a vaginal swab obtained from a healthy woman. The total genome size is 1,806,242 bp with a G+C content of 40.6%.

## ANNOUNCEMENT

Lactobacillus vaginalis was first isolated and described by Embley and colleagues in 1989 (1), and it has been reported to be one of the predominant species colonizing the vaginal microbiota of healthy women (2). However, the onset of bacterial vaginosis significantly reduces the level of L. vaginalis (3). In this paper, we report the complete draft genome sequence of L. vaginalis strain LMG S-26419, isolated by plating a vaginal swab taken from a healthy woman donor on Rogosa agar medium (BD, Difco, NJ, USA) and incubated under anaerobic conditions at 37°C. Sampling was carried out in compliance with the Helsinki Declaration, and an informed written consent was signed. A single isolated colony on Rogosa agar medium (BD, Difco) was submitted for DNA extraction performed with a Whatman CloneSaver card system (96-well format) (VWR, PA, USA) and processed following the manufacturer’s instructions. The P0 (5′-GAGAGTTTGATCCTGGCTCAG-3′) (4) and P2 (5′-GGTGAGTAACGCGTGGGGAA-3′) (5) primer sets were used for the partial amplification of the 16S rRNA gene that was then sequenced (BMR Genomics, Padua, Italy). The nucleotide sequence was used to search the GenBank database (https://www.ncbi.nlm.nih.gov/) using BLAST (6), the blastn algorithm, and the Ribosomal Database Project (7).

For total DNA extraction, a single colony of L. vaginalis was inoculated in MRS-Cys (De Man Rogosa Sharpe BD, Difco) supplemented with 0.5 g/liter of cysteine (Sigma-Aldrich, MO, USA) under anaerobic conditions for 18 h at 37°C, and 1 ml of the liquid culture was aliquoted for the DNA extraction using the MasterPure Gram-positive DNA purification kit (Epicentre, Cambio, UK) following the manufacturer’s instructions. The genomic library was constructed using the Nano-DNA Illumina kit, and the Illumina MiSeq v3 platform for 2 × 300-bp paired-end reads was used to sequence the complete genome (Fasteris, Switzerland). A total of 1,695,030 high-quality reads (i.e., with a Phred score above 30) were obtained after sequencing and quality filtering. KmerGenie v1.6213 software (8) was applied to determine the most appropriate number of k-mers for the assembly, which was then performed with Velvet v1.2.10 (9). Assembly metrics were evaluated with QUAST v4.0 (10), highlighting an *N*_50_ value of 47,415 bp and a largest contig of 1,879,874 bp. Assembly resulted in 123 contigs; the genome has a total length of 1,879,874 bp with a coverage of 80× and a G+C content of 40.6%. Rapid Annotations using Subsystems Technology (RAST) (11) functional annotation of the genome resulted in 1,801 coding sequences and 73 combined rRNAs and tRNAs. The genome was interrogated for the presence of antibiotic resistance genes through the ResFinder database (12), and no positive significant hits were found. Bacteriocin analysis was conducted with BAGEL4 (13), which identified a potential region of interest with two uncharacterized proteins (YqbO and BasT) whose roles as bacteriocins still need to be confirmed.

A BLAST search of the genome sequence of L. vaginalis LMG S-26419 against the LAB-Secretome database (14) resulted in 144 positive hits, which indicates a number of features of potential interest listed in Table 1.

**TABLE 1:** Relevant genes of the *L. vaginalis* LMG S-26419 secretome

Category	Gene ID no.^*a*^	Gene function
Cell wall and capsule	PROKKA_01729	Putative glycosyltransferase EpsH
	PROKKA_00446	Putative sugar transferase EpsL
	PROKKA_00596	Putative glycosyltransferase EpsJ
Adhesion	PROKKA_00005	Putative mucus binding protein
	PROKKA_00115	MucBP domain protein
	PROKKA_00581	MucBP domain protein
	PROKKA_01171	MucBP domain protein
	PROKKA_01173	MucBP domain protein
Glycerol metabolism	PROKKA_01202	Glycerol-3-phosphate dehydrogenase
Lipoteichoic acid metabolism	PROKKA_00017	Lipoteichoic acid synthase
	PROKKA_00368	Lipoteichoic acid synthase
	PROKKA_00431	Lipoteichoic acid synthase
	PROKKA_00571	d-Alanyl-lipoteichoic acid biosynthesis protein DltD
	PROKKA_00573	d-Alanyl-lipoteichoic acid biosynthesis protein DltB
	PROKKA_00804	Lipoteichoic acid protein A
Peroxidase production	PROKKA_00726	NADH oxidase
	PROKKA_00956	NADH flavine reductase

a ID, identification.

### Data availability.

The whole-genome shotgun project has been deposited in NCBI under the BioProject accession number PRJNA504605 with the corresponding GenBank accession number RJTI00000000 and the SRA accession number SRR8206196.
